# MicroRNA hsa-miR-134 is a circulating biomarker for mesial temporal lobe epilepsy

**DOI:** 10.1371/journal.pone.0173060

**Published:** 2017-04-06

**Authors:** Simoni H. Avansini, Beatriz Pereira de Sousa Lima, Rodrigo Secolin, Marilza L. Santos, Ana Carolina Coan, André S. Vieira, Fábio R. Torres, Benilton S. Carvalho, Marina K. M. Alvim, Márcia E. Morita, Clarissa L. Yasuda, Luciana R. Pimentel-Silva, Danyella B. Dogini, Fabio Rogerio, Fernando Cendes, Iscia Lopes-Cendes

**Affiliations:** 1 Department of Medical Genetics, University of Campinas - UNICAMP, and the Brazilian Institute of Neuroscience and Neurotechnology (BRAINN), Campinas, São Paulo, Brazil; 2 Department of Neurology, University of Campinas - UNICAMP, and the Brazilian Institute of Neuroscience and Neurotechnology (BRAINN), Campinas, São Paulo, Brazil; 3 Department of Statistics, Institute of Mathematics, Statistics and Scientific Computing, University of Campinas - UNICAMP, and the Brazilian Institute of Neuroscience and Neurotechnology (BRAINN), Campinas, São Paulo, Brazil; 4 Department of Anatomical Pathology, University of Campinas - UNICAMP, and the Brazilian Institute of Neuroscience and Neurotechnology (BRAINN), Campinas, São Paulo, Brazil; University of Modena and Reggio Emilia, ITALY

## Abstract

Epilepsy is misdiagnosed in up to 25% of patients, leading to serious and long-lasting consequences. Recently, circulating microRNAs have emerged as potential biomarkers in a number of clinical scenarios. The purpose of this study was to identify and to validate circulating microRNAs that could be used as biomarkers in the diagnosis of epilepsy. Quantitative real-time PCR was used to measure plasma levels of three candidate microRNAs in two phases of study: an initial discovery phase with 14 patients with mesial temporal lobe epilepsy (MTLE), 13 with focal cortical dysplasia (FCD) and 16 controls; and a validation cohort constituted of an independent cohort of 65 patients with MTLE and 83 controls. We found hsa-miR-134 downregulated in patients with MTLE (p = 0.018) but not in patients with FCD, when compared to controls. Furthermore, hsa-miR-134 expression could be used to discriminate MTLE patients with an area under the curve (AUC) of 0.75. To further assess the robustness of hsa-miR-134 as a biomarker for MTLE, we studied an independent cohort of 65 patients with MTLE, 27 of whom MTLE patients were responsive to pharmacotherapy, and 38 patients were pharmacoresistant and 83 controls. We confirmed that hsa-miR-134 was significantly downregulated in the plasma of patients with MTLE when compared with controls (p < 0.001). In addition, hsa-miR-134 identified patients with MTLE regardless of their response to pharmacotherapy or the presence of MRI signs of hippocampal sclerosis. We revealed that decreased expression of hsa-miR-134 could be a potential non-invasive biomarker to support the diagnosis of patients with MTLE.

## Introduction

The diagnosis of epilepsy is currently based on neurological history, EEG and neuro-imaging findings [1]. Although with very well defined parameters it may still present a challenge in more complex patients since it also requires a certain degree of clinical experience for the interpretation of the findings in the context of single patients [2]. The correct diagnosis of epilepsy would allow patients to receive an appropriate treatment and could prevent unnecessary side effects from long-term medication such as adverse psychological and social consequences. However, misdiagnosis of epilepsy is frequent, occurring in around 25% of patients [3] and in paediatric series this number is higher; 39% of children in Denmark do not receive correct diagnosis [4]. Furthermore, the estimated cost of wrong diagnoses of epilepsy could reach more than £100,000,000 per year in England [5]. Therefore, there is still the need for additional biomarkers which could improve and support the diagnosis of epilepsy [6, 7], and for better defining cohorts for clinical trials.

Circulating microRNAs are emerging as candidates for use as biomarkers in a number of disorders, ranging from cancer (e.g., miR-21 in breast cancer) [8] to coronary artery disorders (e.g., miR-155) [9]. These are small non-coding RNA molecules (~20 nucleotides) present in extracellular human body fluids, including plasma or serum. Currently, it is well known that induced changes of microRNAs levels are stable in plasma and can be strongly associated with specific disease states [10]. Moreover, circulating microRNA measurement is a non-invasive and easily quantifiable procedure [11].

To our knowledge there are only four reports investigating serum levels of microRNAs in patients with epilepsy as compared to healthy controls [12–15]. Given the great heterogeneity of epilepsy syndromes it becomes clear that additional studies are urgently needed.

In this context, the main goal of this study is to identify and validate a robust and non-invasive biomarker to assist in the diagnosis of epilepsy. We quantified plasma levels of three candidate microRNAs, previously associated with epilepsy: hsa-miR-134 [16] reported in temporal lobe epilepsy (TLE); hsa-miR-31 [17], identified by our group in brain tissue from patients with FCD, and hsa-miR-23a [18] identified in rat models of TLE.

## Materials and methods

### Patients and study design

We recruited patients from 2013 to 2015 at the outpatient epilepsy clinic of the University of Campinas (UNICAMP) hospital, which is a tertiary centre for epilepsy care. Prior to undergo any study procedures all patients and control subjects signed a written informed consent. The Comitê de Ética em Pesquisa from the University of Campinas specifically approved this study (CAAE: 12112913.30000.5404).

The clinical evaluation of patients was performed by neurologists with experience in the treatment of patients with epilepsy. All patients were interviewed using a structured questionnaire gathering information regarding age, onset of epilepsy (OSF), history of febrile seizure (FS), family history of epilepsy (FH) and number of AEDs used. In addition, all patients underwent a neurological exam, serial interictal EEGs and high resolution MRI with a specific epilepsy protocol. Hippocampal atrophy and other MRI signs of hippocampal sclerosis (HS) were assessed by visual analyses and the images were classified as having normal findings or signs of HS. Patients with dual pathology or tumours were not included. Clinical characteristics are summarised in Table 1. Controls individuals were Brazilian unrelated individuals, with no neurological or psychiatric disease, who voluntarily agreed to donate plasma samples to our study.

**Table 1:** Clinical findings in patients with FCD, MTLE and control individuals enrolled in both phases of the study.

Discovery cohort					
Variable		FCDType II(n = 13)	MTLE(n = 14)	Controls(n = 16)	p-value
Sex	Male	10	6	6	0.104
	Female	3	8	10	
OSF	Yes	12	6	-	0.100
	No	1	8	-	
HS	Yes	0	14	-	**1.504e-06**
	No	13	0	-	
FS	Yes	2	3	-	1
	No	11	11	-	
FH	Yes	5	7	-	0.83
	No	8	7	-	
Average number of AEDs used		5.8	4.15	-	0.60
Seizure frequency (monthly)*		157	12.1	-	**2.2e-16**
Validation cohort					
		MTLE AED Responsive(n = 27)	MTLE AED Resistant (n = 38)	Controls (n = 83)	p-value
Sex	Male	12	16	35	1
	Female	15	22	48	
OSF	Yes	11	14	-	0.952
	No	16	24	-	
HS	Yes	16	32	-	**0.049**
	No	11	6	-	
FS	Yes	6	9	-	1
	No	21	29	-	
FH	Yes	12	14	-	0.72
	No	15	24	-	
Average number of AEDs used		2.53	4.97	-	0.37
Seizure frequency (monthly)*		0	6.35	-	**0.012**

FCD: Focal Cortical Dysplasia; MTLE: Mesial Temporal Lobe Epilepsy AED: antiepileptic drug; OSF: Onset of seizures in the1^st^ decade of life; HS: MRI sings of hippocampal sclerosis; FS: Antecedent of febrile seizure; FH: Presence of family history of epilepsy. χ^2^ test, Yates correction, p<0.05.

* Complex focal seizures and generalized tonic-clonic seizure

The study was performed in two phases: an initial discovery phase and a subsequent validation phase. First, we enrolled 14 patients with MTLE classified according to clinical, electroencephalographic and MRI criteria [19]; 13 patients with focal cortical dysplasia (FCD) type II confirmed by histopathology [20] as well as 16 healthy control subjects, without epilepsy.

Subsequently, in a validation phase, we enrolled an additional independent cohort of 65 patients with MTLE using the same diagnostic criteria as described above. These patients were subsequently divided in two groups according to their response to AED treatment and seizure frequency: i) drug-responsive MTLE (n = 27), defined as seizure freedom for at least 24 months and ii) drug-resistant MTLE (n = 38), defined as any frequency of seizures in the last 24 months, after the trial of at least two AEDs at optimal doses. We also recruited an additional 83 healthy individuals without epilepsy as a control group.

### Blood collection and RNA isolation

For plasma preparation, we collected peripheral blood (4 ml) into EDTA tubes held on ice for up to three hours. The tubes were subjected to centrifugation at 515 x g for 10 min, 4°C. Next, 1 ml aliquots of the plasma were transferred to 1.5 ml tubes and centrifuged at 12,000 x g for 10 min, 4°C to pellet any remaining cellular debris. Subsequently, the supernatant was transferred to fresh tubes and stored at -80°C. The concentration of free haemoglobin was measured in patient plasma by spectrophotometric (BioTek Instruments, Inc., Winooski, EUA) method and samples with A_414_ reading > 0.2 were excluded. MirVana PARIS microRNA Isolation kit was used for extraction of the enriched fraction of small RNAs according to the manufacturer instructions (Ambion Inc, Austin, USA). The final elution volume was 35ul RNase-free water. The concentration and purity were analysed using spectrophotometer.

### Reverse transcription and quantitative real-time PCR

A fixed volume of 5 ul of small RNA-enriched fraction was used as an input into a reverse transcription (RT) reaction using TaqMan^™^ reverse transcription kit (Life Technologies, Foster City, USA) with microRNA specific stem-loop primers, following the manufacturer instructions. The primers used were: hsa-miR-23a (ID399), hsa-miR-31 (MIMAT0000089, ID 2279) and hsa-miR-134 (MIMAT0000447, ID 1186) as candidate microRNAs; hsa-miR-16 (MIMAT0000069, ID391), hsa-miR-191(MIMAT0000440, ID 2299), hsa-miR-451 (MIMAT0001631, ID 1141), RNU24 (ID 1001), and RNU48 (ID1006) as endogenous controls (Life Technologies). We selected hsa-miR-191and hsa-miR-451 for endogenous controls as both are expressed at high levels in plasma and they were relatively stable across samples from patients and control individuals. Data was analyzed with the DataAssist^™^ Software (Life Technologies). The threshold cycle (Cq) values were determined using automatic baseline settings. All reactions were performed in triplicate. The relative quantification was calculated with 2^-ΔΔCt^ method after normalization to hsa-miR-191 and hsa-miR-451. MicroRNAs with an expression level lower than the threshold value (Cq≥36) and with a detection rate below 75% in both group samples were considered rejected. MicroRNA expression levels (row data) as well clinical information of patients included in this study are available in the supporting material; this data is part of our relevant minimal data set (S1 Table).

### Statistical analysis

We used the R statistical environment (version 3.1.2 (2014-10-31) [21] for statistical analysis. Since microRNA expression levels did not follow a normal distribution, we performed a logarithmic transformation. The level of significance, alpha, was set to ≤ 0.05 for log2 transforms of relative expression (RQ), which corresponds to 2^-ΔΔCt^ Bonferroni-adjusted p-values. We used two-sample t-*tests* to perform comparisons of log2 (RQ) between the available groups for each of the three microRNAs. The comparisons of interest were: in the discovery phase, FCD versus controls, MTLE versus controls and FCD versus MTLE; in the validation phase, MTLE versus controls, AED-responsive versus controls, AED-resistant versus controls, and AED-responsive versus AED-resistant patients.

Sensitivity, specificity and area under the curve (AUC) for specific microRNAs were estimated using receiver operator characteristic (ROC) using the caret R package [22]. We applied the bootstrap resampling strategy to optimize the AUC via the caret package implementation. Different clinical parameters between the groups of patients and controls were compared using chi-squared test with Yates correction.

## Results

**Fig 1:**
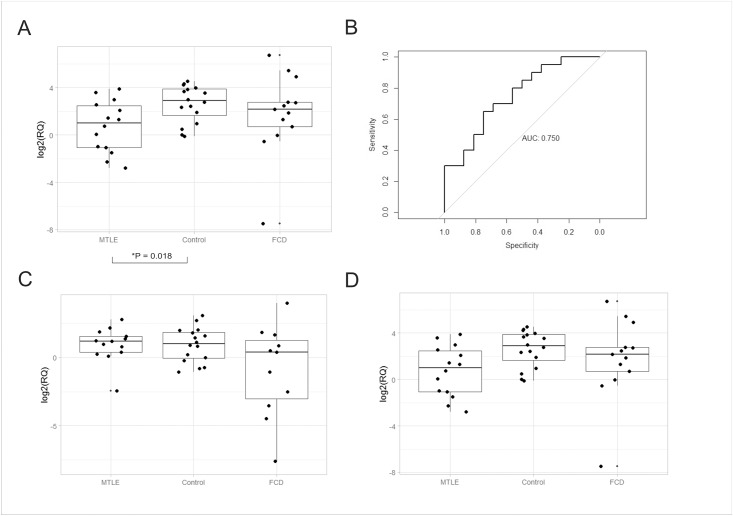
Plasma levels of the three candidates microRNAs quantified in the first cohort of patients (discovery phase). Box plot depicting the log2 transformed relative expression (RQ) of (A) hsa-miR-134 in the three groups, 14 patients with MTLE, 13 patients with FCD and 16 control individuals without epilepsy; (B) receiver-operator curve (ROC) of hsa-miR-134 comparing controls and patients with MTLE. (C) Box plot depicting the log2 transformed RQ values of hsa-miR-23a and (D) hsa-miR-31 in the same three groups. Expression levels were normalized to hsa-miR-191 and hsa-miR-451. The only comparison with statistically significant difference, determined by Student t-test corrected by Bonferroni, is marked with a star (*). Circles indicate outliers.

First, we aimed to determine if differences in microRNA levels are present in patients with epilepsy independent of aetiology. We identified that only hsa-miR-134 was significantly downregulated in plasma of patients with MTLE when compared to controls (p = 0.018; Fig 1A). In addition, plasma levels of this microRNA could discriminate patients with MTLE from control individuals with an AUC of 0.75 with sensitivity of 65% and specificity of 75% in plasma (Fig 1B; 95%CI: 0.589–0.911). However, no difference in plasma levels of hsa-miR-134 was observed in patients with FCD compared to controls (p = 1; Fig 1A; AUC = 0.45). Moreover, expression values of hsa-miR-23a (p _MTLE x Control_ = 1; p _FCD X Control_ = 0.356; p _MTLE X FCD_ = 0.266; Fig 1C) and hsa-miR-31 (p _MTLE x Control_ = 1; p _FCD X Control_ = 1; p _MTLE X FCD_ = 0.88; Fig 1D) were not different among the groups analysed.

**Fig 2:**
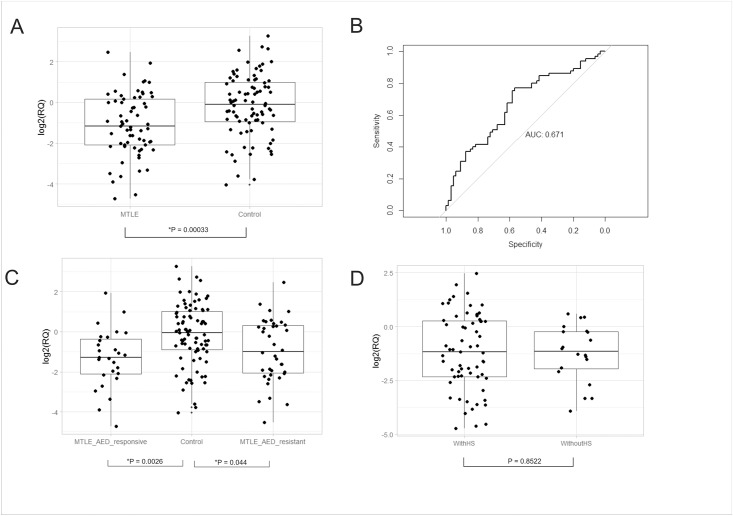
Plasma levels and ROC plot calculated for hsa-miR-134 in the validation cohort. (A) Box-plots depicting log2 transformed RQ values of hsa-miR-134 plasma levels comparing 65 patients with MTLE with 83 control subjects without epilepsy; (B) ROC curve of data shown in (A). (C) Box-plots depicting log2 transformed RQ values of hsa-miR-134 plasma levels comparing 27 patients with AED-responsive MTLE, 83 control subjects and 38 patients with AED-resistant MTLE. (D) Box-plots depicting log2 transformed RQ values of hsa-miR-134 plasma levels comparing patients with MTLE with (n = 48) and without (n = 17) the presence of signs indicating HS on MRI. Expression levels were normalized to hsa-miR-191 and hsa-miR-451. Comparisons with statistically significant differences, determined by the Student t-test corrected by Bonferroni, are marked with stars (*).

Based on these initial results, we decide to focus on hsa-miR-134 in further analyses aiming to verify whether hsa-miR-134 was robust enough to identify patients with MTLE independent of specific clinical characteristics, including response to treatment with AEDs. Therefore, we quantified levels of hsa-miR-134 in the plasma of an additional independent cohort of 65 patients with MTLE. We confirmed that hsa-miR-134 was significantly downregulated in the plasma of these patients when compared with controls without epilepsy (p = 0.00033; Fig 2A). Furthermore, the accuracy for identifying patients with MTLE was AUC = 0.671 with a sensitivity of 75% and a specificity of 58% (Fig 2B; 95%CI: 0.580–0.755). We also showed that hsa-miR-134 is downregulated both in patients with AED-responsive MTLE (p = 0.0026; Fig 2C) and AED-resistant MTLE (p = 0.044; Fig 2C), when compared to control subjects. No difference in hsa-miR-134 plasma levels was observed between AED-responsive and AED-resistant MTLE patients (p = 0.88; Fig 2C). Subsequently, we also evaluated whether signs of HS on MRI could have an impact on hsa-miR-134 plasma levels in patients with MTLE and found no statistical difference between these two groups of (p = 0.8522; Fig 2D). Finally, we investigate if seizure-frequency could affect hsa-miR-134 expression and we used a score of up to one seizure per month to define two groups of patients according to seizure frequency [14] and found no statistical difference between the two groups (p = 0.633; data not shown) regarding plasma levels of hsa-miR-134 plasma.

## Discussion

We investigated plasma levels of three microRNAs in a study designed to identify a non-invasive biomarker that could assist in the sub-syndromic diagnosis of epilepsy. We selected microRNAs hsa-miR-134, hsa-miR-31 and hsa-miR-23a, since they have been reported in abnormal levels in tissue of patients or animal models with different types of epilepsy [16–18]. Our results clearly show a significant downregulation of hsa-miR-134 in plasma of patients with MTLE. In addition, we demonstrated reduced levels of hsa-miR-134 in two independent cohorts of patients with MTLE, regardless their response to treatment with AEDs and presence of MRI signs of HS. The fact that we found no difference in hsa-miR-134 plasma levels between AED-responsive and AED-resistant MTLE patients indicates that hsa-miR-134 plasma levels is not influenced by response to treatment and it is therefore, a biomarker for MTLE and not for response to AED treatment. We found no difference in the levels of any of the microRNAs tested in patients with FCD compared to controls.

Jimenez—Mateos and collaborators [16], in their elegant study, were the first to show that miR-134 is upregulated in both temporal lobe and hippocampal pyramidal neurons of pharmacoresistant patients with TLE. They suggested that dysregulation of this microRNA could be a response to abnormal neuronal activity, potentially associated with alterations in dendritic spines density. Here we show, for the first time, that hsa-miR-134 is present in plasma of patients with MTLE and it is downregulated in comparison to healthy controls, suggesting that hsa-miR-134 could be used as a biomarker for MTLE. Similarly, Takana et al. [23] revealed that plasma miR-92a was found downregulated in non-Hodgkin’s lymphoma compared with healthy subjects, although miR-92a is overexpressed in malignant lymphoma cells. They suggested that microRNAs are packaged inside exosomes that are secreted from cells, but in tumour these exosomes may be encompassed by cancer cells and consequently miR-92a decreases from the blood.

Because 15–38% of patients with MTLE have normal MRI [24, 25], this group of patients may constitute an additional challenge for diagnosis [26]. Thus, the identification of a non-invasive biomarker that could be used to support the diagnosis of MTLE in these patients would be of paramount importance. Our results show that hsa-miR-134 could also be used to support diagnosis of MTLE in patients without MRI signs of HS. Thus, plasma levels of hsa-miR-134 could be used, in addition to other clinical and EEG parameters to help distinguishing between psychogenic non-epileptic seizures (PNES) and epileptic seizures. PNES diagnosis, particularly in individuals with normal MRI, may be lengthy, due to the difficulty and the challenge to establish the correct diagnosis [27, 28] and therefore, patients with PNES could significantly benefit from the use of a minimally invasive biomarker of epileptic seizures. Nevertheless, since we did not directly performed this type of analysis, further studies including patients with PNES should be performed to further explore the usefulness of determining plasma levels of hsa-miR-134 in this specific clinical application.

To date, few studies have addressed the issue of circulating microRNAs as potential biomarkers for epilepsy. An initial study in a rat model of TLE [29], identified differences in plasma levels of miR-21, miR-146 and miR-142 in different phases of the epileptogenic process. More recently, Wang and collaborators [12] published the first report identifying differences in expression levels of circulating microRNAs in serum in a mix group of patients with partial and generalized epilepsy. Subsequently, the same authors [13], studying a clinically heterogeneous group of drug-resistant and drug-responsive patients with idiopathic and cryptogenic epilepsy found abnormal expression levels of hsa-miR-301a as a good candidate to discriminate these two groups. Because of the remarkable heterogeneity in terms of aetiology and underlying mechanisms in different forms of epilepsy, we designed the present study to include well defined epilepsy syndromes aiming to avoid the confounding factors which could hinder the identification of reliable biomarkers in epilepsy [30].

It was recently shown that circulating microRNAs can be deregulated by precedent seizures [14], in order to investigate this issue we used a score of up to one seizure per month to define two groups of patients and found no statistical difference regarding plasma levels of hsa-miR-134 plasma, which further indicates that hsa-miR-134 plasma levels are indeed stable across a number of potential confounding variables. We are also aware that AEDs may have an effect on circulating microRNAs [31]. Unfortunately, our sample size is rather small due to the high variability of antiepileptic drug treatments and daily drug dose. However, since we found no difference in hsa-miR-134 plasma levels between AED-responsive and AED-resistant MTLE patients, which have clear differences in AED daily doses as well as AED-regimen, we believe this is an indication that hsa-miR-134 plasma levels may not suffer significant changes due to the effect of different AEDs.

Although our results can be considered of marginal statistical significance, they indicate the need for additional large studies, ideally including different patients with different types of epilepsy syndromes, seizure frequency and AED-regimen. In addition, it is more realistic to assume that no single biomarker will attain 100% sensitivity of specificity and that a combination of different biomarkers together with clinical information is more likely to be used in clinical practice.

In conclusion, we showed that decreased expression of hsa-miR-134 could be a potential and non-invasive biomarker to support the diagnosis of patients with MTLE. In addition, we have presented evidence supporting our findings in two independent cohorts of patients with MTLE. Therefore, we suggest that the determination of hsa-miR-134 plasma levels could represent a valuable tool to support the diagnosis of patients with MTLE in conjunction with clinical, EEG, and imaging parameters, pending additional confirmatory studies.

## Supporting information

S1 Table MicroRNAs expression and clinical findings in patients with FCD, MTLE and control individuals enrolled in discovery and validation phases. (XLSX)

## References

[1] Shorvon S D. The etiologic classification of epilepsy. Epilepsia. 2011; 52: 1052–1057. 10.1111/j.1528-1167.2011.03041.x 21449936

[2] Moshé SL, Perucca E, Ryvlin P, Tomson T. Epilepsy: new advances. Lancet. 2015; 385(9971): 884–898. 10.1016/S0140-6736(14)60456-6 25260236

[3] Ferrie C D. Preventing misdiagnosis of epilepsy. Arch Dis Child. 2006; 91(3): 206–209. 10.1136/adc.2005.088906 16492881 PMC2065943

[4] Uldall P, Alving J, Hansen L K, Kibæk M, Buchholt J.The misdiagnosis of epilepsy in children admitted to a tertiary epilepsy centre with paroxysmal events. Arch Dis Child. 2006; 91(3): 219–221. 10.1136/adc.2004.064477 16492886 PMC2065931

[5] Juarez-Garcia A, Stokes T, Shaw B, Camosso—Stefinovic J, Baker R. The costs of epilepsy misdiagnosis in England and Wales. Seizure. 2006;15(8):598–605. 10.1016/j.seizure.2006.08.005 17011217

[6] Mathern GW. Challenges in the surgical treatment of epilepsy patients with cortical dysplasia. Epilepsia 2009; 50 Suppl 9:45–50. 10.1111/j.1528-1167.2009.02294.x 19761453

[7] Engel J, Pitkänen A, Loeb JA, Dudek FE, Bertram EH, Cole AJ, et al Epilepsy biomarkers. Epilepsia. 2013; 54 Suppl 4:61–69. 10.1111/epi.12299 PMC4131763 23909854

[8] Yan LX, Huang XF, Shao Q, Huang MY, Deng L, Wu QL, et al. MicroRNA miR-21 overexpression in human breast cancer is associated with advanced clinical stage, lymph node metastasis and patient poor prognosis. RNA. 2008;14:2348–60. 10.1261/rna.1034808 18812439 PMC2578865

[9] Fichtlscherer S, De Rosa S, Fox H, Schwietz T, Fischer A, Liebetrau C, et al. Circulating microRNAs in patients with coronary artery disease. Circ Res. 2010;107(5):677–84. 10.1161/CIRCRESAHA.109.215566 20595655

[10] Schwarzenbach H, Nishida N, Calin GA, Pantel K. Clinical relevance of circulating cell-free microRNAs in cancer. Nat Rev Clin Oncol. 2014; 11:145–156 10.1038/nrclinonc.2014.5 24492836

[11] Mitchell PS, Parkin RK, Kroh EM, Fritz BR, Wyman SK, Pogosova-Agadjanyan EL, et al Circulating microRNAs as stable blood-based markers for cancer detection. Proc Natl Acad Sci USA. 2008; 105:10513–10518. 10.1073/pnas.0804549105 18663219 PMC2492472

[12] Wang J(a), Yu JT, Tan L, Tian Y, Ma J, Tan CC, et al Genome-wide circulating microRNA expression profiling indicates biomarkers for epilepsy.Sci. Rep. 2015b;5:9522. 25825351 10.1038/srep09522 PMC4379481

[13] Wang J(b), Tan L, Tan L, Tian Y, Ma J, Tan CC et al Circulating microRNAs are promising novel biomarkers for drug-resistant epilepsy. Sci Rep. 2015;5:10201–10210. 10.1038/srep10201 25984652 PMC4435024

[14] Surges R, Kretschmann A, Abnaof K, van Rikxoort M, Ridder K, Fröhlich H, et al Changes in serum miRNAs following generalized convulsive seizures in human mesial temporal lobe epilepsy. Biochem Biophys Res Commun. 2016;481:13–18 10.1016/j.bbrc.2016.11.029 27833019

[15] Sun J, Cheng W, Liu L, Tao S, Xia Z, Qi L, et al Identification of serum miRNAs differentially expressed in human epilepsy at seizure onset and post-seizure. Mol Med Rep. 2016:5318–5324. 10.3892/mmr.2016.5906 27840934

[16] Jimenez-Mateos EM, Engel T, Merino-Serrais P, McKiernan RC, Tanaka K, Mouri G, et al Silencing microRNA-134 produces neuroprotective and prolonged seizure-suppressive effects. Nat Med 2012;18:1087–1094. 10.1038/nm.2834 22683779 PMC3438344

[17] Avansini SH, Torres FR, Dogini DB, Rogerio F, Coan AC, Secolin R. et al. Dysfunction of miRNAs biogenesis in association with neuronal differentiation in Focal Cortical Dysplasia. http://www.ashg.org/2012meeting/abstracts/fulltext/f120121920.htm

[18] Song YJ, Tian XB, Zhang S, Zhang YX, Li X, Li D, et al. Temporal lobe epilepsy induces differential expression of hippocampal miRNAs including let-7e and miR-23a/b. Brain Res. 2011;1387:134–140. 10.1016/j.brainres.2011.02.073 21376023

[19] Berg AT, Berkovic SF, Brodie MJ, Buchhalter J, Cross JH, van Emde Boas W, et al Revised terminology and concepts for organization of seizures and epilepsies: report of the ILAE Commission on Classification and Terminology, 2005–2009. Epilepsia. 2010;51:676–685. 10.1111/j.1528-1167.2010.02522.x 20196795

[20] Blümcke I, Thom M, Aronica E, Armstrong DD, Vinters HV, Palmini A, et al The clinicopathologic spectrum of focal cortical dysplasias: a consensus classification proposed by an ad hoc Task Force of the ILAE Diagnostic Methods Commission. Epilepsia. 2011; 52:158–174. 10.1111/j.1528-1167.2010.02777.x 21219302 PMC3058866

[21] R Core Team (2014). R: A language and environment for statistical computing. R Foundation for Statistical Computing, Vienna, Austria URL http://www.R-project.org/.

[22] Simon N, Friedman J, Hastie T, Tibshirani R. Regularization Paths for Cox's Proportional Hazards Model via Coordinate Descent. J Stat Softw. 2011; 39(5), 1–13. 10.18637/jss.v039.i05 27065756 PMC4824408

[23] Tanaka M, Oikawa K, Takanashi M, Kudo M, Ohyashiki J, Ohyashiki K, et al Down-regulation of miR-92 in human plasma is a novel marker for acute leukemia patients. PLoS One. 2009;4:e5532 10.1371/journal.pone.0005532 19440243 PMC2678255

[24] Jackson GD, Kuzniecky RI, Cascino GD. Hippocampal sclerosis without detectable hippocampal atrophy. Neurology. 1994;44:42–46. 8290088 10.1212/wnl.44.1.42

[25] Coan AC, Kubota B, Bergo FP, Campos BM, Cendes F. 3T MRI quantification of hippocampal volume and signal in mesial temporal lobe epilepsy improves detection of hippocampal sclerosis. AJNR Am J Neuroradiol. 2014;35:77–83. 10.3174/ajnr.A3640 23868151 PMC7966486

[26] Carne RP, O'Brien TJ, Kilpatrick CJ, MacGregor LR, Hicks RJ, Murphy MA et al. MRI-negative PET-positive temporal lobe epilepsy: a distinct surgically remediable syndrome. Brain. 2004;127:2276–85. 10.1093/brain/awh257 15282217

[27] de Timary P, Fouchet P, Sylin M, Indriets JP, de Barsy T, Lefèbvre A, et al Non-epileptic seizures: delayed diagnosis in patients presenting with electroencephalographic (EEG) or clinical signs of epileptic seizures. Seizure. 2002;11:193–197. 10.1053/seiz.2001.0617 12018963

[28] Bodde NM, Brooks JL, Baker GA, Boon PA, Hendriksen JG, Aldenkamp AP. Psychogenic non-epileptic seizures—diagnostic issues: a critical review. Clin Neurol Neurosurg 2009;111:1–9. 10.1016/j.clineuro.2008.09.028 19019531

[29] Gorter JA, Iyer A, White I, Colzi A, van Vliet EA, Sisodiya S, et al. Hippocampal subregion-specific microRNA expression during epileptogenesis in experimental temporal lobe epilepsy. Neurobiol. Dis. 2014; 62: 508–520. 10.1016/j.nbd.2013.10.026 24184920

[30] Pitkänen A, Löscher W, Vezzani A, Becker AJ, Simonato M, Lukasiuk K, et al Advances in the development of biomarkers for epilepsy. Lancet Neurol. 2016;15(8):843–56. 10.1016/S1474-4422(16)00112-5 27302363

[31] de Boer HC, van Solingen C, Prins J, Duijs JM, Huisman MV, Rabelink TJ, et al Aspirin treatment hampers the use of plasma microRNA-126 as a biomarker for the progression of vascular disease. Eur Heart J. 2013;34:3451–3457. 10.1093/eurheartj/eht007 23386708

